# A Deep Learning Framework with Qualitative Post Hoc Explanation for Dry Eye Detection and Tear Film Lipid Layer Pattern Classification Using Anterior-Segment and Tearscope Imaging

**DOI:** 10.3390/diagnostics16213569

**Published:** 2026-10-29

**Authors:** Sajedeh Talebi, Neda Abdolvand, Mohammad Hassan Emamian, Akbar Fotouhi, Hassan Hashemi, Sara Mazaheri

**Affiliations:** 1 Department of Management, Faculty of Social Sciences and Economics, Alzahra University, Tehran 1993893973, Iran; sajedeh.talebi1998@gmail.com (S.T.); s.mazaheri@alzahra.ac.ir (S.M.); 2 Ophthalmic Epidemiology Research Center, Shahroud University of Medical Sciences, Shahroud 3614773955, Iran; emamian@shmu.ac.ir; 3 Department of Epidemiology and Biostatistics, School of Public Health, Tehran University of Medical Sciences, Tehran 1985717413, Iran; afotouhi@tums.ac.ir; 4 Noor Ophthalmology Research Center, Noor Eye Hospital, Tehran 1968653111, Iran; hhashemi@norc.ac.ir

**Keywords:** dry eye disease, tear film lipid layer, Guillon patterns, Tearscope, ResNet transfer learning, post hoc explanation, Grad-CAM

## Abstract

**Background/Objectives:** Dry eye disease (DED) is assessed by combining patient-reported symptoms with ocular surface findings. We evaluated a deep learning strategy with qualitative post hoc explanation in two independent ophthalmic imaging tasks: DED-versus-healthy classification from anterior segment slit lamp images; and four-class recognition of tear film lipid layer (TFLL) interference patterns in Tearscope images using the Guillon categories. **Methods:** ResNet backbones were modified by adding parallel expansion channels while keeping the ImageNet-initialized channels frozen. The added channels were He-normal initialized and trained at 1 × 10^−4^, with the MLP classification head trained at 1 × 10^−3^. Latin hypercube sampling was used for hyperparameter selection within the development data. We used Grad-CAM and Grad-CAM++ only for post hoc interpretation. We evaluated ShECS slit lamp images (*n* = 434) and VOPTICAL-Is Tearscope images (*n* = 406). **Results:** Under the original evaluations, ResNet-101×3 achieved the highest scores among the evaluated configurations on both tasks. On ShECS, accuracy was 74% (95% *CI*: 0.63–0.82), with precision 0.80, recall 0.90, and *F1*-score 0.85 for the DED (positive) class, and AUC 0.80. On VOPTICAL-Is, the original image-level evaluation yielded four-class TFLL accuracy of 90% (95% *CI*: 0.82–0.95), with macro precision 0.89, macro recall 0.90, and macro F14 0.89, and a macro one-versus-rest AUC of 0.98. **Conclusions:** These preliminary proof-of-concept results support further evaluation of the combined channel-expansion and differential-training strategy, together with qualitative post hoc explanation methods for ophthalmic image classification. Because VOPTICAL-Is contains only healthy-subject images and has no documented subject identifiers in the accessed release (group-code overlap between the development and held-out subsets was observed, and subject overlap cannot be excluded), the second task represents image-level TFLL pattern classification rather than clinical DED-subtype diagnosis. A separate code-grouped sensitivity analysis with a frozen ResNet-50 feature extractor yielded 0.59 held-out accuracy (macro one-versus-rest AUC 0.85). Because this grouped run did not re-run the 0.90 ResNet-101×3 configuration, the numerical difference cannot be attributed solely to partitioning, or interpreted as a direct measurement of subject-level leakage; rather, it demonstrates sensitivity to group-aware partitioning. The original image-level estimates should therefore be treated as exploratory and not as evidence of subject-level generalization. A controlled 2 × 2 factorial ablation (channel width × trainability policy) on the same code-grouped partition, executed as a TensorFlow/Keras ResNet-50 reconstruction with three seeds per cell, indicated descriptively that unfreezing the final convolutional stage was associated with higher accuracy and macro F1 (e.g., standard width: accuracy 0.556 vs. 0.469), whereas the effect of widening was small and inconsistent across metrics and policies. These descriptive summaries involve no significance testing and do not measure subject-level leakage. External clinical validation of identified cohorts is needed before routine use.

## 1. Introduction

Dry eye disease (DED) reflects a loss of tear film homeostasis, and can involve aqueous-deficient and evaporative mechanisms that frequently coexist [1,2,3,4]. Estimates of prevalence vary with the population studied and the diagnostic definition applied [4,5,6]. Because symptoms and objective signs do not always coincide, clinical assessment generally combines both, rather than relying on a single test [7]. Identifying the dominant mechanism can also influence management choices [8]. Tearscope imaging provides a view of tear film lipid layer (TFLL) interference patterns; the Guillon categories include open meshwork, closed meshwork, wave, and color fringe [9,10,11,12]. These patterns describe the tear film image and should not be equated with patient-level DED phenotypes such as aqueous-deficient, evaporative, or mixed-disease. We therefore examined two independent image-classification tasks: DED versus healthy-eye classification from ShECS anterior segment slit lamp images; and four-class TFLL pattern recognition from VOPTICAL-Is Tearscope images. The modeling workflow combines channel-widened CNN backbones, transfer learning, hyperparameter optimization, and post hoc explanation. Recent DED imaging studies have used deep learning with videokeratography, fluorescein-stained slit lamp images, and related ocular imaging approaches, while reviews continue to emphasize external validation and clinically robust evaluation as major needs [13,14,15,16]. The individual components used here (ResNet backbones, ImageNet transfer learning, channel widening, Latin hypercube sampling, and Grad-CAM/Grad-CAM++) are established, and we do not claim a new backbone or optimizer. The contribution of this study lies in its specific, reproducible combination and its preliminary evaluation in two small-sample ophthalmic imaging settings: (i) widening ResNet backbones with trainable, He-normal-initialized channels concatenated to frozen ImageNet channels, with separate learning rates for the expansion channels and the classification head; (ii) space-filling hyperparameter selection confined to the development partition with repeated cross-validation; along with (iii) an explicit account of the partitioning constraints, including the absence of subject identifiers in VOPTICAL-Is. The results are intended as preliminary proof-of-concept evidence and not as evidence of clinical diagnostic performance. Clinical interpretation of the VOPTICAL-Is task as DED diagnosis or subtyping, as well as any deployment, remains outside the scope of the present study and requires separate prospective validation.

## 2. Materials and Methods

Two independently trained classification branches were implemented within a common experimental workflow. The ShECS branch distinguished DED from healthy eyes using anterior segment slit lamp images, whereas the VOPTICAL-Is branch assigned one of four Guillon TFLL interference pattern categories to Tearscope images. The second branch is an image-pattern classifier and not a clinical DED-subtype classifier. To accommodate the modest dataset sizes, images were prepared consistently, and ResNet channel width was expanded threefold (fourfold for ResNet-152). Pretrained ImageNet channels were kept fixed; in addition, the added channels were He-normal initialized and optimized at 1 × 10^−4^, while the classification head was optimized at 1 × 10^−3^. Hyperparameter selection was restricted to the development data. Grad-CAM and Grad-CAM++ were generated only after classification for qualitative interpretation, and the held-out test sets were not used during model selection. 

**Figure 1:**
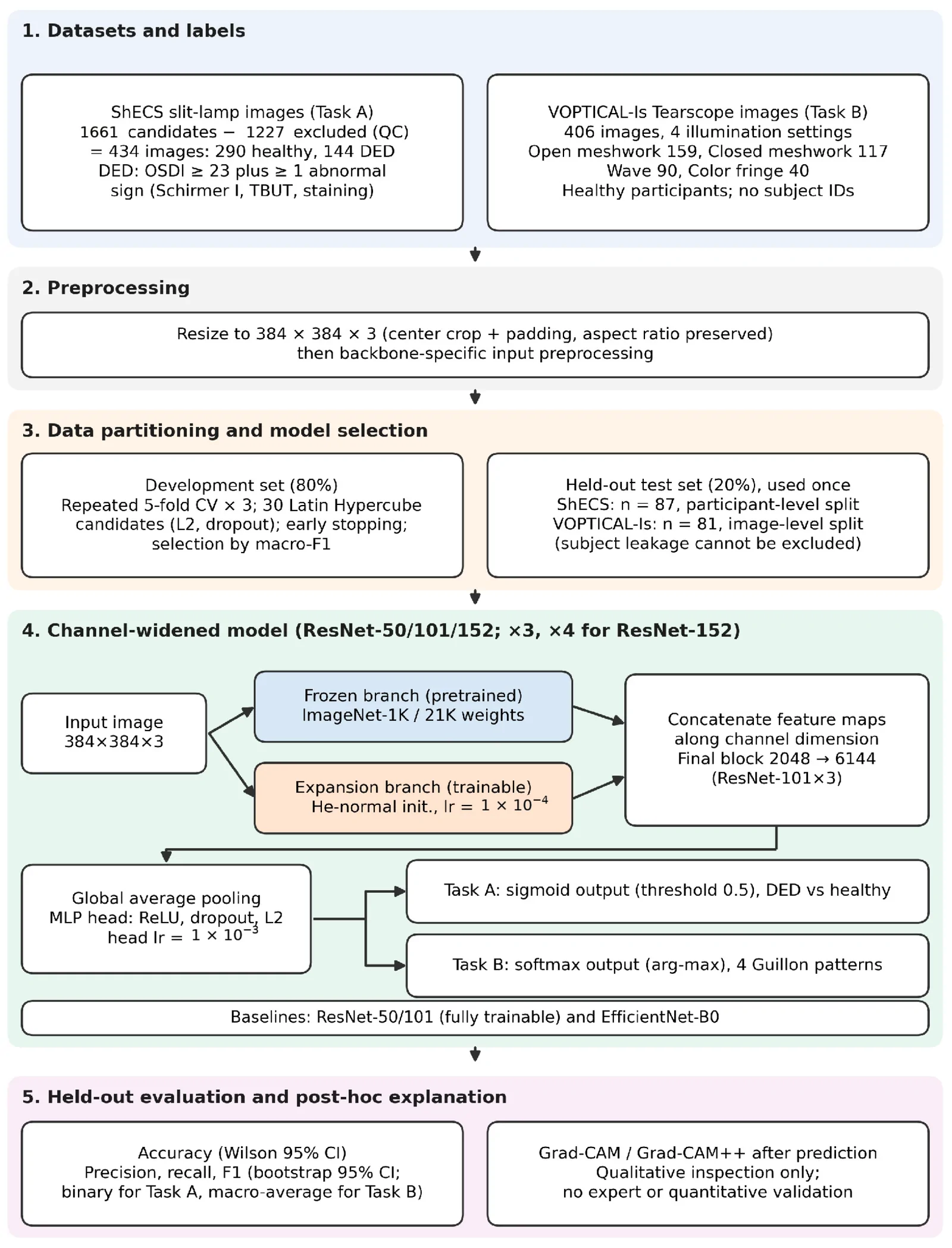
Detailed workflow of the study. (1) Dataset characteristics and labeling; (2) preprocessing; (3) partitioning into a development set (repeated cross-validation with Latin hypercube hyperparameter search) and a held-out test set; (4) channel-widened architecture with a frozen pretrained branch, a trainable He-normal expansion branch, channel-wise concatenation, global average pooling, and an MLP head (Task A, DED versus healthy; Task B, four TFLL patterns); (5) held-out evaluation and qualitative post hoc explanation. Pastel background bands distinguish the five workflow stages.

Figure 1 summarizes the experimental workflow. Each dataset underwent its own quality-control and partitioning steps, followed by resizing, backbone-specific preprocessing, feature extraction, LHS-based tuning, and final evaluation on a held-out test set. The ShECS branch predicts DED versus healthy eye, whereas the VOPTICAL-Is branch assigns one of four Guillon TFLL patterns. No patient-level fusion was performed across the two datasets. Grad-CAM and Grad-CAM++ were produced after inference to inspect model attention. Any teleophthalmology use would require additional engineering and clinical validation.

### 2.1. Data Collection and Preparation

ShECS is a population-based eye cohort conducted in Shahroud, Iran [17]. The present analysis used images from its first phase in 2009. The slit-lamp images were acquired using a Topcon SL-7E Photo slit lamp (Topcon Corporation, Tokyo, Japan). Starting from 1661 candidate images, quality review removed 1227 because of closed eyes, eyelash or eyelid obstruction, invalid imaging devices, black or red backgrounds, or resource-related error, the latter affecting 14 images. The resulting analytical set contained 434 images: 290 healthy-eye images and 144 DED images. All images analyzed here were de-identified. DED labels were assigned according to the ShECS dry eye assessment protocol [18]. Symptom burden was measured with the Ocular Surface Disease Index (OSDI), and objective assessment included Schirmer I testing, tear break-up time (TBUT), fluorescein corneal staining, and Rose Bengal staining. A DED label required an OSDI score ≥ 23 together with at least one abnormal sign: Schirmer I < 10 mm/5 min, TBUT < 10 s, or positive ocular surface staining. Controls had OSDI < 23 and no abnormal objective test.

VARPA distributes the VOPTICAL-Is (VOPTICAL_Is) image set used for the second task [9,19]. The accessed release contains 406 preocular TFLL images obtained under four illumination settings from healthy, dark-eyed participants aged 19–33 years. The images were acquired using a Tearscope Plus (Keeler Ltd., Windsor, UK) attached to a Topcon SL-D4 slit lamp and a Topcon DV-3 digital video camera (Topcon Medical Systems, Oakland, NJ, USA), and were stored using Topcon IMAGEnet i-base software (Topcon Medical Systems, Oakland, NJ, USA; version not reported in the source documentation) at a spatial resolution of 1024 × 768 pixels in RGB. Class counts are 159 open meshwork, 117 closed meshwork, 90 wave, and 40 color fringe [19]. VOPTICAL-Is does not contain an amorphous class; that category belongs to the separate VOPTICAL_I1-v2 variant [19]. Acquisition and labeling were performed by optometrists from the Optometry Service at the University of Santiago de Compostela, Spain [19]. VARPA requests that users of the datasets cite the dataset and the associated publications [9,19]. Accordingly, this part of the study concerns four-class TFLL image pattern recognition rather than clinical DED subtype diagnosis. Images from both datasets were resized to 384 × 384 × 3 using center cropping and padding to preserve aspect ratio. Backbone-specific preprocessing was applied immediately before transfer learning. ShECS was partitioned at participant level so that both eyes of individual participants remained in the same subset. VOPTICAL-Is used a stratified image-level 80:20 split because subject identifiers are not documented in the accessed release. Repeated 5-fold cross-validation with three repetitions was confined to the 80% development portion; the 20% test portion was used only after model selection. Because subject grouping is not documented for VOPTICAL-Is, leakage related to repeated images from the same subject cannot be excluded. The original VOPTICAL methodological study reports that its image banks were acquired from the same group of healthy participants, and illustrates two images from the same subject [9]. The VARPA dataset documentation lists no participant identifiers [19]. Multiple correlated images from one participant may therefore be present in VOPTICAL-Is. For ResNet backbones, tf.keras.applications.resnet.preprocess_input converted RGB input to BGR and subtracted the ImageNet channel means without [0, 1] rescaling. EfficientNet-B0 used the preprocessing built into tf.keras.applications.EfficientNetB0. tf.keras.applications.efficientnet.preprocess_input is a backward-compatibility pass-through, with input rescaling handled inside the model. Post hoc, we noticed that the image file names contain a three-digit code (001–016) that may correspond to participants; this was not documented, and could not be confirmed. In our image-level split, all test images shared this code with training images (100% across 20 random 80:20 splits). As a diagnostic, a simple non-deep baseline (logistic regression on 16 × 12 RGB thumbnails plus channel means and standard deviations) reached 0.83 accuracy under repeated stratified image-level 5-fold cross-validation, but reached only 0.40 when folds were grouped by this code; in addition, it predicted the code itself with 0.97 accuracy (16 classes). These observations motivated a stricter code-grouped sensitivity analysis but did not establish that the code is a subject identifier. A code-grouped 80:20 partition was constructed at the group level, with the constraints that approximately 20% of images be held out and that all four Guillon categories be represented in the held-out subset; the four held-out codes (002, 005, 011, 016; 81 images) and the complete file-level assignment of all 406 images are provided in Supplementary Material. This partition was used for a code-grouped sensitivity analysis with a frozen ResNet-50 feature extractor and a retrained multilayer-perceptron head, using the same preprocessing and optimization framework as the original workflow (ImageNet initialization, seed 42). Because the grouped run did not reproduce an identical original configuration under both partitioning strategies, it was used to assess sensitivity to group-aware partitioning rather than to quantify subject-level leakage or a partition-only effect. The analysis is reported in Section 4.2. To isolate the effects of channel width and backbone trainability under this same partition, an additional controlled 2 × 2 factorial ablation was executed in TensorFlow/Keras with a reconstructed ResNet-50 backbone: channel width (standard vs. widened ×3) was crossed with trainability policy (frozen backbone vs. trainable final-stage conv5 blocks). Specifically, in the two frozen cells the pretrained ResNet-50 backbone was kept frozen and the classification head was trained, with the trainable expansion branch additionally included in the widened/frozen cell; in the two conv5-trainable cells the final conv5 stage of the backbone was set trainable in addition to the classification head (and the expansion branch in the widened/conv5-trainable cell), so that the trainability factor refers to the backbone freezing policy rather than to all non-head components. The partition, preprocessing, optimization budget, and held-out images were held fixed (293 training, 32 validation, and 81 held-out test images; the same 81-image held-out set across all seeds; three seeds per cell). A 79-image sensitivity set retaining one deterministic representative per exact-duplicate image pair was evaluated in parallel. Because the original custom training source is unavailable, this reconstruction is not an exact source-code re-execution of the original custom-widened ResNet pipeline; the ablation results are reported in Section 4.2. For reproducibility, the reconstruction is specified as follows: in the widened cells, the 2048-channel final-stage representation of the ResNet-50 backbone was expanded to 6144 channels by a trainable, He-normal-initialized expansion branch running in parallel to the frozen pretrained branch, and the two branches were concatenated along the channel dimension before global average pooling applied at the final residual stage only; the classification head was a single-hidden-layer multilayer perceptron with ReLU, dropout, and L2 regularization (256 hidden units, dropout 0.30, L2 1 × 10^−4^); and training used Adam with a batch size of 32, a 100-epoch maximum, and early stopping after 10 epochs without improvement in validation loss with best-weights restoration, with the classification head at 1 × 10^−3^ and the expansion branch at 1 × 10^−4^. The trainable conv5 stage used Adam at 1 × 10^−5^.

### 2.2. Model Architecture

ResNet backbones obtained through TensorFlow/TensorFlow Hub [20] and EfficientNet-B0 [21] were initialized with ImageNet-1K or ImageNet-21K weights according to the implementations used in the experiments. The widened ResNets tripled channel width, with the exception of ResNet-152 which used a fourfold expansion. Pretrained channels were preserved and frozen, while added filters were initialized with He-normal (Kaiming-normal) initialization [22] and optimized with a smaller learning rate. The classification stage used global average pooling followed by a single-hidden-layer multilayer perceptron (MLP) [23]. ReLU, dropout, and L2 regularization were applied in the hidden layer; the binary DED branch used sigmoid output, and the four-class TFLL branch used softmax. The classification head and expansion filters were trained at 1 × 10^−3^ and 1 × 10^−4^, respectively. LHS determined the dropout and L2 settings, and no data augmentation was applied. Experiments were run with TensorFlow 2.15.0; TensorFlow Hub 0.16.1 supplied the corresponding pretrained components. Feature-extraction handles were loaded from TensorFlow Hub. For ResNet-101×3 and ResNet-152×4; the pretrained branch remained non-trainable, and the He-normal expansion branch was trainable. A custom Keras module concatenated their feature maps along the channel dimension:

*H_widened_* = *Concat*(*f_frozen_*(*X*; *W_base_*), *f_trainable_*(*X*; *W_added_*))

Backpropagation updated only the expansion branch (learning rate 1 × 10^−4^) and the final MLP head (learning rate 1 × 10^−3^); the pretrained backbone was excluded from gradient updates. Total and trainable parameter counts were checked with model.summary() before the experiments. We use the standard ResNet residual notation [24]:

*y* = *F*(*x*;{*W_i_*}) + *x*

Here, *F*(.) Fdenotes the residual transformation, *x* is the block input, and *y* is the block output. When the dimensions of *x* and *F*(*x*) differ, the shortcut uses a projection matrix *W*_s_:

*y* = *F*(*x*;{*W_i_*}) + *W_s_x*

In the widened models, each residual representation combines the frozen pretrained channels with the trainable expansion channels. Added filters were He-normal initialized [22] and optimized at 1 × 10^−4^, whereas the ImageNet-derived filters remained fixed. Thus, the final residual block of ResNet-101×3 increased from 2048 to 6144 filters. EfficientNet-B0 [21] served as a non-widened baseline. Global average pooling transformed the final convolutional tensor into a feature vector *z* before the MLP head.

$$z_{k}=\frac{1}{\mathrm{HW}}\sum_{i=1}^{H}\sum_{j=1}^{W}A_{\mathrm{ij}}^{k}$$

*k* = 1,…,*C*

The pooled representation is:

$$z={\left[z_{1},\;z_{2},\ldots ,\;z_{c}\right]}^{T}$$

Here, $A_{\mathrm{ij}}^{k}$ is the activation in channel k at spatial location (*i*, *j*); *H* and *W* are the feature-map height and width, and *C* is the channel count after widening (*C* = 6144 for ResNet-101×3). The pooled vector is then passed to the one-hidden-layer MLP with ReLU, dropout, and L2 regularization.

*h* = *Dropout*(*ReLU*(*W*_1_*z* + *b*_1_),*p*)

The binary branch converts the MLP representation to a DED probability using a sigmoid function:

$$P_{\mathrm{DED}}=\sigma \;\left(w_{\mathrm{DED}}^{T}h\right)+b_{\mathrm{DED}}$$

The TFLL branch maps the same representation to four class probabilities using softmax:

*P_TFLL_* = *softmax*(*W_TFLL_h* + *b_TFLL_*)

In these expressions, W1 and b1 are the hidden-layer parameters; *W_DED_* and *b_DED_* are the binary output parameters; and *W_TFLL_* and *b_TFLL_* are the four-class output weight matrix and bias vector. The head was optimized at 1 × 10^−3^ and the expansion filters at 1 × 10^−4^, while pretrained ImageNet channels remained frozen. LHS selected dropout probability *p* and L2 strength. A sigmoid value of 0.5 defined the DED decision threshold for ShECS. For VOPTICAL-Is, the predicted TFLL label was the class with the largest softmax probability. Grad-CAM and Grad-CAM++ were calculated after prediction and did not alter class assignment.

### 2.3. Hyperparameter Optimization and Internal Validation

**Figure 2:**
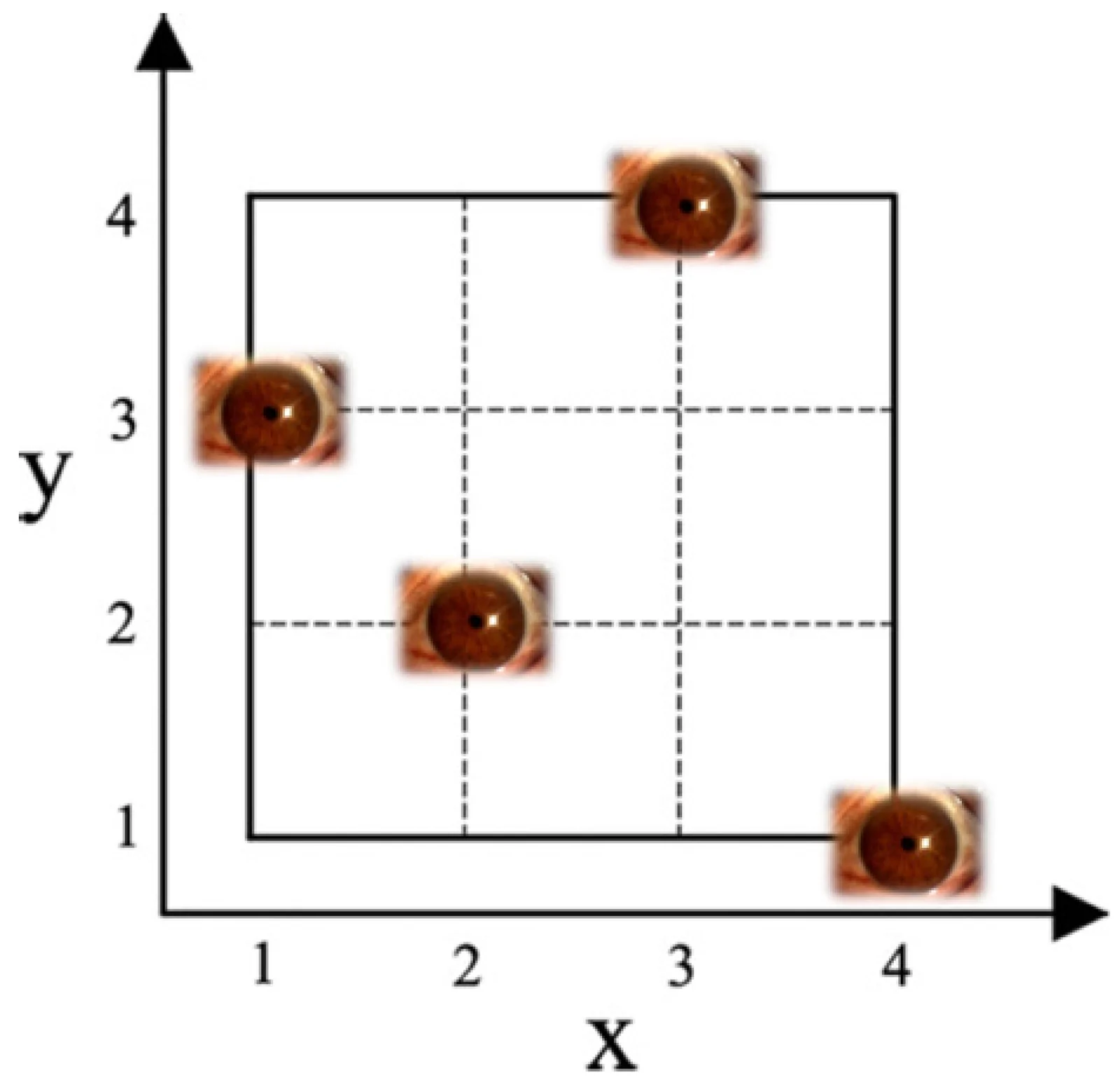
Two-dimensional illustration of the Latin hypercube hyperparameter design.

The search space covered L2 strength (0.0001–0.01) and dropout probability *p* (0.1–0.5); the learning rates were fixed (1 × 10^−3^ for the MLP head and 1 × 10^−4^ for the expansion channels), and the number of epochs was governed by early stopping. Latin hypercube sampling (LHS) [25] was used to draw space-filling candidate configurations. All tuning was carried out inside the 80% development partition using 5-fold cross-validation repeated three times; the held-out 20% portion was not used for model selection. Figure 2 shows a two-parameter projection of the LHS design.

Figure 2 visualizes lambda and *p* as one slice of the multidimensional LHS. Variation in the reported metrics across the repeated folds remained below 2%; we interpret this only as evidence of internal stability of the selection procedure. Thirty LHS candidate vectors were evaluated [25]. With three repetitions and five folds, this produced 30 × 3 × 5 = 450 model fits for each candidate architecture. Adam optimization used a batch size of 32 and a 100-epoch limit. Binary cross-entropy was used for ShECS, and categorical cross-entropy for VOPTICAL-Is. Training was stopped after 10 epochs without improvement in validation loss, and the best weights were restored. The configuration with the highest validation macro-F1 was selected. Accuracy confidence intervals were computed with the Wilson method. Precision, recall, *F1*-score, and AUC are reported as point estimates on the held-out predictions (Section 2.5). The code was developed interactively in Python/Jupyter on a local machine, while model training and inference ran on the central high-performance server at Shahroud University of Medical Sciences. All computational experiments were conducted using Python 3.11.5 and Jupyter Notebook 6.5.4. Deep-learning models were implemented using PyTorch 2.14.0 and Torchvision 0.29.0. Model interpretability was performed using Gradient-weighted Class Activation Mapping (Grad-CAM) and Grad-CAM++, implemented with the grad-cam (pytorch-grad-cam) package, version 1.5.7. Grad-CAM and Grad-CAM++ were used to generate class-specific activation maps highlighting image regions that contributed to the model’s predictions.

### 2.4. Qualitative Post Hoc Explanation

**Figure 3:**
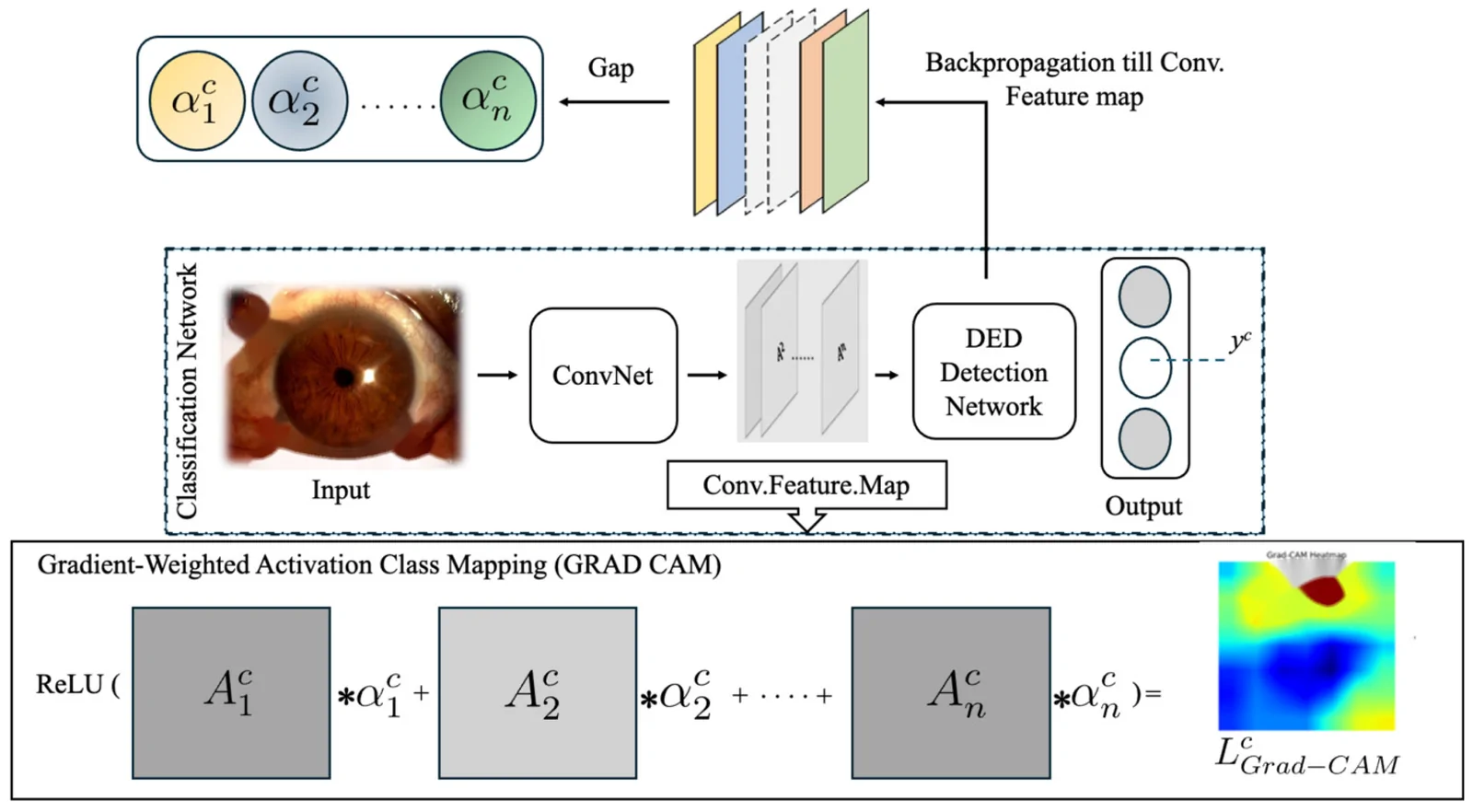
Post hoc explanation workflow for Grad-CAM and Grad-CAM++.

Qualitative post hoc explanations were generated with Grad-CAM [26] and Grad-CAM++ [27] for visual inspection only. Grad-CAM weights the final convolutional feature maps using gradients of the target pre-softmax score, whereas Grad-CAM++ uses location-specific weights derived from higher-order derivatives. Heatmaps were resized to the input dimensions and superimposed on the source images (Figure 3). They were inspected only qualitatively; no comparison with expert-delineated regions or quantitative localization score was carried out.

Figure 3 shows the explanation calculation. For a selected class c, a forward pass through ResNet-101×3 yields the pre-softmax score *S^c^*S^c and the final convolutional maps *A^k^*A^k. Let $A_{\mathrm{ij}}^{k}$A^k_ij denote the activation at location (i, j) in map k. The Grad-CAM channel coefficient is:

$$\alpha_{k}^{c}=\;\frac{1}{\mathrm{HW}}\;\sum_{i=1}^{H}\sum_{j=1}^{W}\frac{\partial S^{c}}{\partial A_{\mathrm{ij}}^{k}}$$

With *H* × *W* denoting the spatial size of the final convolutional feature maps, the class-specific Grad-CAM map2 is:

$$L_{\mathrm{Grad}\_\mathrm{CAM}}^{c}=\mathrm{ReLU}\left(\sum_{k}\alpha_{k}^{c}A^{k}\right)$$

The resulting map was interpolated to input resolution and overlaid on the original image. Grad-CAM++ instead assigns a position-dependent coefficient within each feature map. Using the target logit *S^c^*, the smooth score function is defined as:

*Y^c^* = exp(*S^c^*)

Higher-order derivatives of *Y^c^* determine the location-specific Grad-CAM++ coefficients:

$$\alpha_{\mathrm{ij}}^{\mathrm{kc}}=\;\frac{\frac{\partial^{2}Y^{c}}{{\left(\partial A_{\mathrm{ij}}^{k}\right)}^{2}}}{2\frac{\partial^{2}Y^{c}}{{\left(\partial A_{\mathrm{ij}}^{k}\right)}^{2}}+\sum_{a}\sum_{b}A_{\mathrm{ab}}^{k}\frac{\partial^{3}Y^{c}}{{\left(\partial A_{\mathrm{ij}}^{k}\right)}^{3}}}$$

These coefficients are aggregated to obtain the class-specific weight for feature map k:

$$W_{k}^{c}=\;\sum_{i}\sum_{j}\alpha_{\mathrm{ij}}^{\mathrm{kc}}\;\mathrm{ReLU}\;\left(\frac{\partial Y^{c}}{\partial A_{\mathrm{ij}}^{k}}\right)$$

The Grad-CAM++ localization map is then obtained as:

$$L_{\mathrm{Grad}-\mathrm{CAM}++}^{c}=\mathrm{ReLU}\;(\sum_{k}w_{k}^{c}A^{k})$$

Because Grad-CAM++ incorporates second- and third-order derivative terms, it can distribute class evidence more locally than Grad-CAM. In this study both methods were used solely for qualitative visual inspection of where the trained models concentrated predictive evidence. Any apparent emphasis on tear-meniscus or lipid-interference regions was not quantified, and was not compared with expert-delineated regions; therefore, this must not be interpreted as validated localization or clinical explainability.

### 2.5. Evaluation

Performance was evaluated separately for the ShECS DED task and the VOPTICAL-Is four-class TFLL task. We report accuracy, precision, recall, *F1*-score, and area under the ROC curve (AUC) [28]. DED was treated as the positive class in the binary analysis.

$$\mathrm{Accuracy}=\frac{\mathrm{True}\;\mathrm{Positives}+\mathrm{True}\;\mathrm{Negatives}}{\mathrm{Total}\;\mathrm{Predictions}}$$

$$\mathrm{Precision}=\frac{\mathrm{True}\;\mathrm{Positives}}{\mathrm{True}\;\mathrm{Positives}+\mathrm{False}\;\mathrm{Positives}}$$

$$\mathrm{Recall}=\frac{\mathrm{True}\;\mathrm{Positives}}{\mathrm{True}\;\mathrm{Positives}+\mathrm{False}\;\mathrm{Negatives}}$$

$$F1-\mathrm{Score}=2.\;\frac{\mathrm{Precision}.\mathrm{Recall}}{\mathrm{Precision}+\mathrm{Recall}}$$

For VOPTICAL-Is, precision, recall, and *F1*-score were calculated one-versus-rest for each Guillon category and then averaged across the four categories:

$$\mathrm{Macro}-\mathrm{Precision}=\;\frac{1}{4}\sum_{c=1}^{4}{\mathrm{Precision}}_{c}$$

$$\mathrm{Macro}-\mathrm{Recall}=\frac{1}{4}\sum_{c=1}^{4}{\mathrm{Recall}}_{c}$$

$$\mathrm{Macro}-F1=\frac{1}{4}\sum_{c=1}^{4}{F1}_{c}$$

Here, c indexes the four TFLL categories. Macro averaging gives each category equal weight regardless of its sample count. Accuracy uncertainty was summarized with a 95% Wilson score interval:

$${\mathrm{CI}}_{95\%}=\;\frac{p+\;\frac{z^{2}}{2n}+z\sqrt{\frac{p(1-p)}{n}}+\frac{z^{2}}{4n^{2}}}{1+\frac{z^{2}}{n}}$$

Here, *p* is the observed test accuracy, *n* is the number of test images, and *z* = 1.96 for a two-sided 95% interval. Precision, recall, *F1*-score, and AUC are reported as point estimates on the held-out test sets. AUC was computed from the held-out predicted probabilities: with DED as the positive class for the binary task, and as the macro average of the four one-versus-rest AUCs for the four-class task. For the code-grouped sensitivity analysis, aggregate results are reported in the main text. The ablation’s training/validation loss and accuracy curves, 4 × 4 held-out confusion matrices, and one-versus-rest ROC curves are provided in Supplementary Figures S1–S4; all twelve per-seed metric records are reported in Supplementary Table S1. The eleven original configurations were not re-executed under the code-grouped partition; accordingly, no group-aware curves or configuration rankings are inferred for those original image-level runs.

## 3. Results

Results are reported separately for the ShECS binary task and the VOPTICAL-Is four-class TFLL task. Table 1 and Table 2 summarize the original held-out evaluations. ShECS used a participant-level split, as shown in Table 1. Table 2 reports the original VOPTICAL-Is image-level split. The separate code-grouped sensitivity analysis is reported in Section 4.2, and should not be interpreted as an identical re-run of the Table 2 configurations. For VOPTICAL-Is, values for precision, recall, and *F1*-score are macro averages across open meshwork, closed meshwork, wave, and color fringe. The controlled 2 × 2 factorial ablation on the code-grouped partition is also reported in Section 4.2.

On ShECS, ResNet-101×3 ranked first among the tested configurations: accuracy 0.74 (95% *CI*: 0.63–0.82), precision 0.80, recall 0.90, and *F1*-score 0.85 for the DED (positive) class, with AUC 0.80; identical results were obtained for the ImageNet-1K and ImageNet-21K initializations of this configuration.

Table 2 gives the corresponding VOPTICAL-Is results (image-level TFLL pattern classification on healthy-subject images). Accuracy is overall image-level accuracy, whereas precision, recall, and *F1*-score are the arithmetic means of the four class-specific one-versus-rest values.

**Figure 4:**
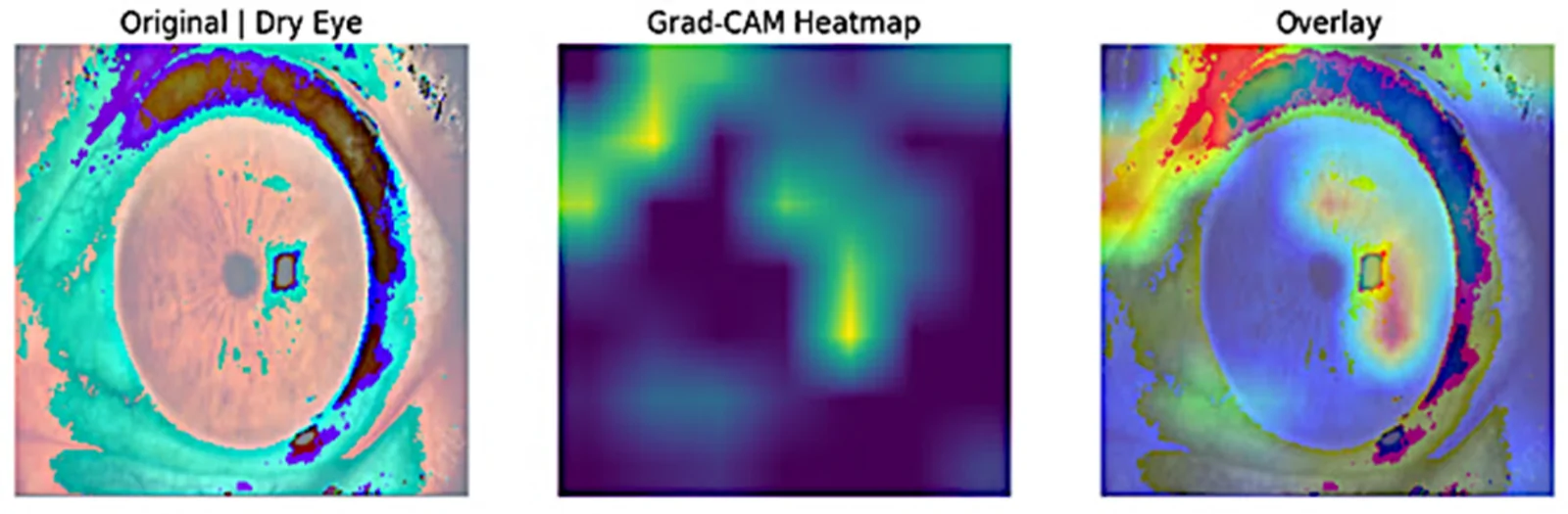

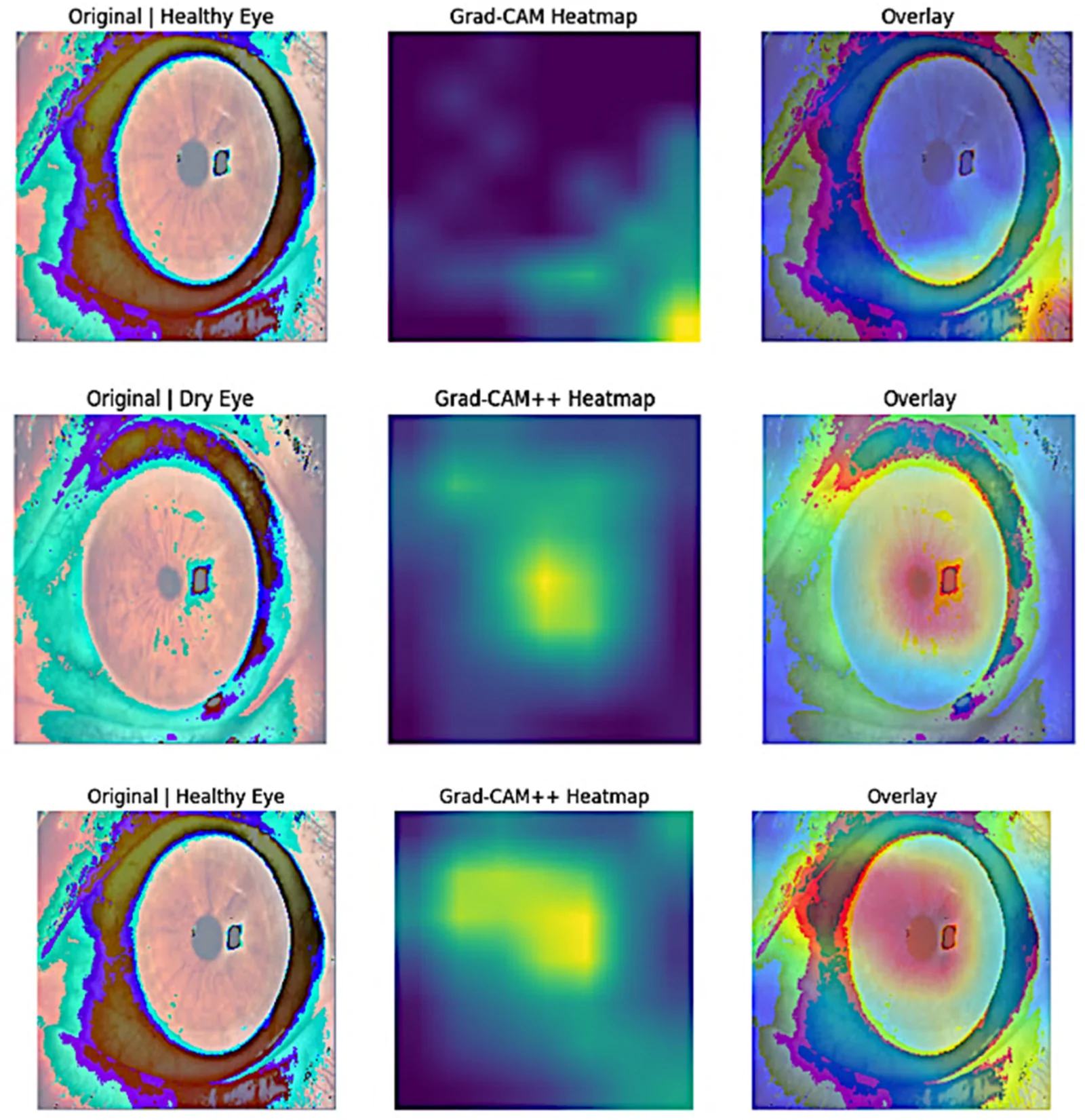
Grad-CAM and Grad-CAM++ responses for the ShECS binary DED classifier. Heatmap colors indicate relative model activation, with warmer colors showing stronger responses and cooler colors showing weaker responses.

**Figure 5:**
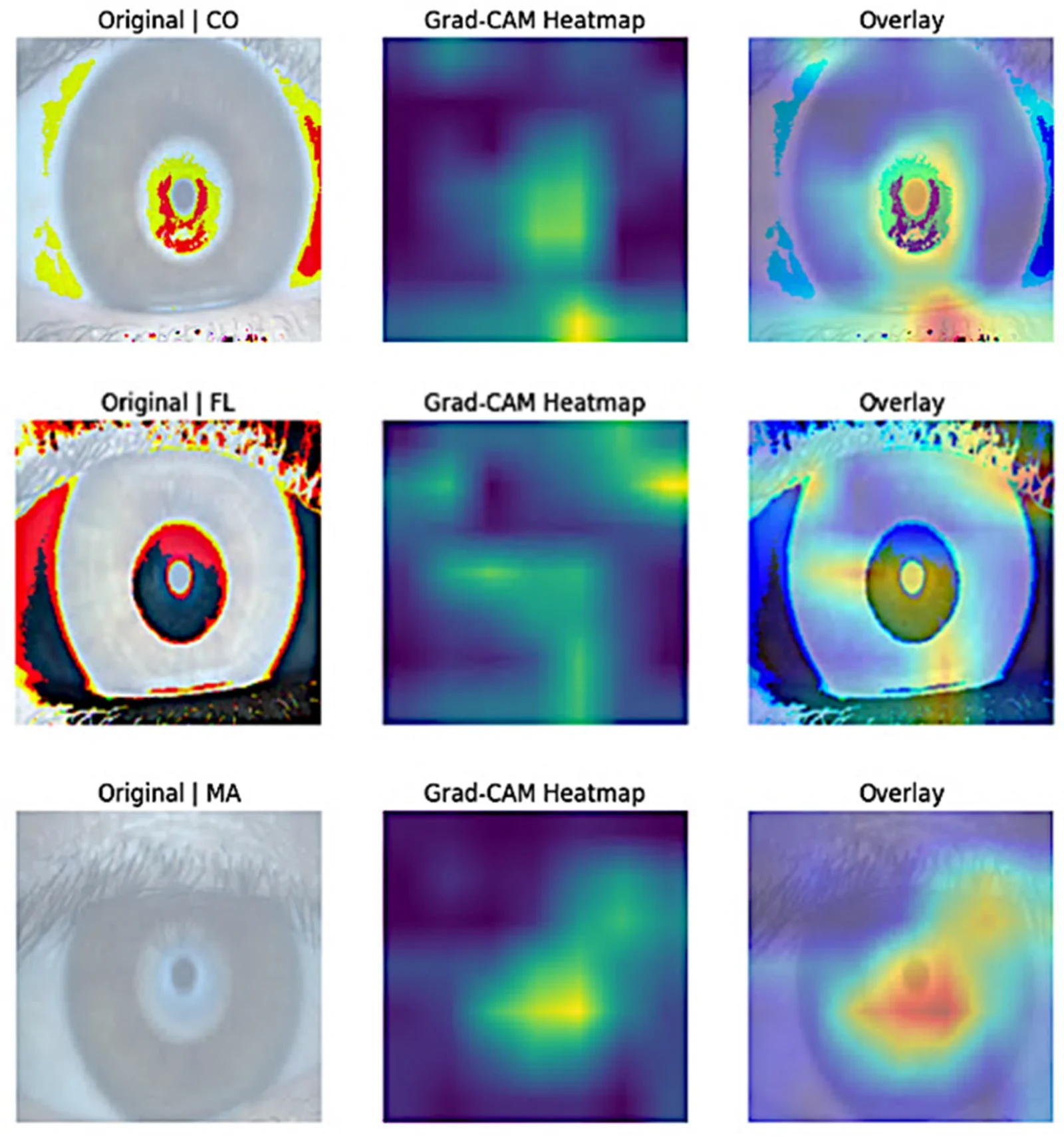

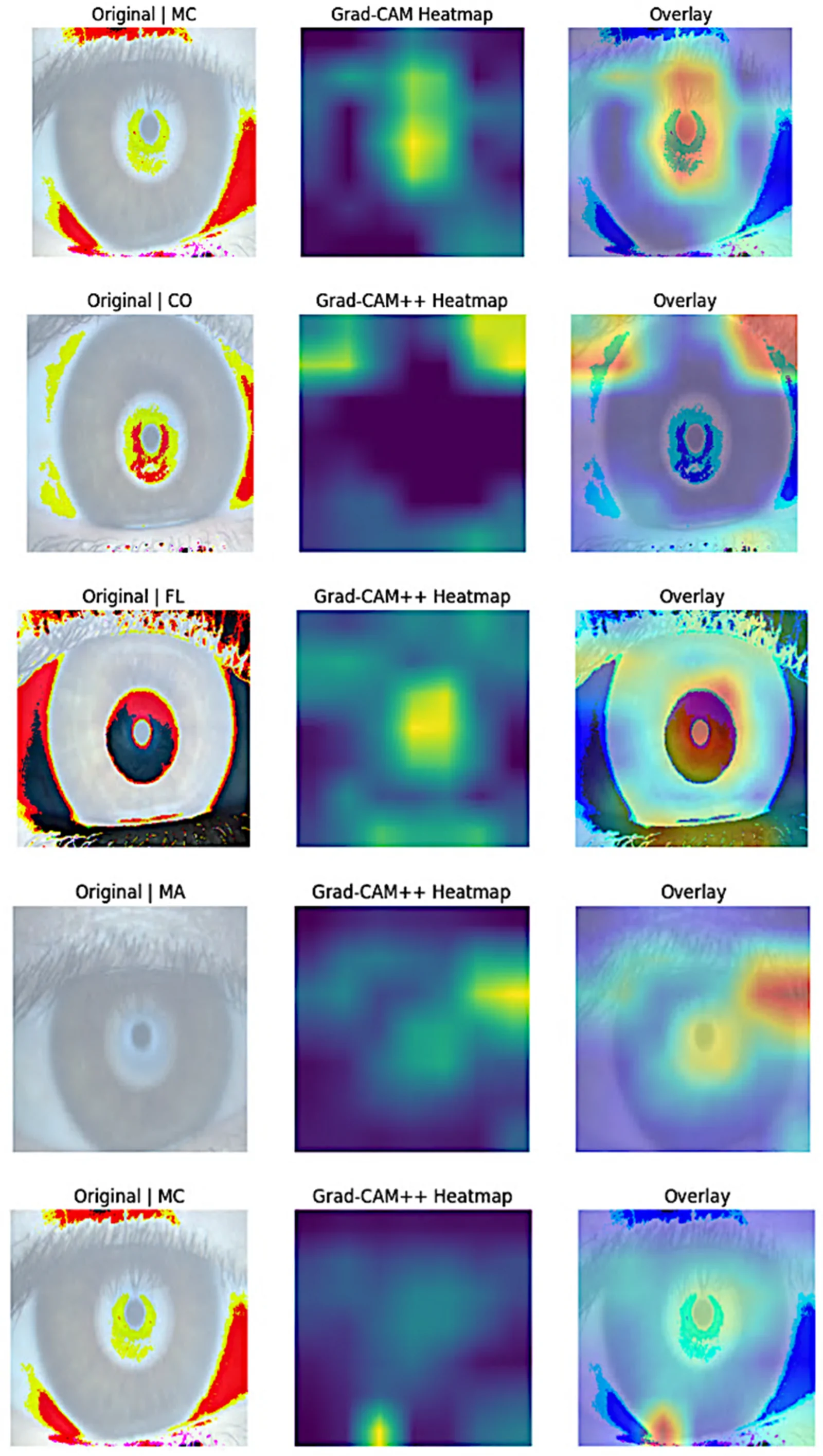
Grad-CAM and Grad-CAM++ responses for the four VOPTICAL-Is TFLL categories. Heatmap colors indicate relative model activation, with warmer colors showing stronger responses and cooler colors showing weaker responses.

Within the original image-level VOPTICAL-Is comparison, ResNet-101×3 with ImageNet-1K weights had the highest numerical accuracy, reaching 0.90 (95% *CI*: 0.82–0.95), with macro precision 0.89, macro recall 0.90, macro F1 0.89, and macro one-versus-rest AUC 0.98 on the four-class TFLL pattern task (healthy subjects only). This ranking applies only to the original image-level split: the eleven configurations were not re-executed under the code-grouped partition, so no configuration ranking is claimed for group-aware or subject-level generalization. Figure 4 and Figure 5 show qualitative Grad-CAM/Grad-CAM++ maps from the highest-performing configuration within the original image-level evaluation. These maps were generated for visual inspection only and were not scored against expert-drawn regions.

## 4. Discussion

On ShECS, ResNet-101×3 achieved the highest held-out accuracy among the evaluated configurations. On VOPTICAL-Is, ResNet-101×3 had the highest numerical accuracy within the original image-level comparison, but that ranking has not been established under code-grouped evaluation. These findings are best regarded as preliminary proof-of-concept results. The experiments combined channel expansion, differential freezing/training, transfer learning, LHS-based tuning, and qualitative post hoc explanation. Importantly, the widened models differed from the fully trainable baselines in both architecture and freezing/training policy; the observed image-level performance differences therefore reflect the combined design and cannot be attributed to channel width alone without a controlled ablation. ShECS and VOPTICAL-Is also cannot be treated as an external validation pair: although they originate from Iran and Spain, they use different imaging modalities, populations, and prediction endpoints. Consequently, Table 3 only compares the TFLL component with earlier VOPTICAL family studies [9,10,11,12,29] and with one recent TFLL interferometry study [30].

Recent DED-related imaging research has approached the problem from several directions, including videokeratography-based subtype classification [13] and automated grading of fluorescein-stained slit lamp images [14]. Reviews of this literature identify heterogeneous datasets and limited external validation as continuing barriers to clinical translation [15,16]. Explainability tools are increasingly reported in other ophthalmic classifiers as well [31], but heatmaps do not replace prospective or task-specific clinical validation.

### 4.1. Comparison with Related Studies

Relative to earlier VOPTICAL-family studies, the TFLL branch (image-level pattern classification on healthy-subject images) achieved a similar performance range while replacing hand-engineered color/texture descriptors with learned CNN features and adding qualitative post hoc explanation maps. Cross-study percentages should not be compared as if they came from the same experiment. Table 3 lists the accuracy or classification rate, the measure common to these studies as summarized here, and the differences in partitioning protocols across studies (for example, 10-fold cross-validation in [10], a random 50:50 partition in [29], and a stratified image-level 80:20 split in the present study). A more recent lightweight-CNN study that combined EfficientNet-B0 and MobileNetV2 features with metaheuristic feature selection and a KNN classifier [29] reported 98.08% and 98.15% accuracy on VOPTICAL_I1 and VOPTICAL_L. These are variants other than VOPTICAL_Is, and they were evaluated under a different partition; those figures are therefore not directly comparable with ours. A recent smartphone interferometry study [30] classified image patches from one healthy participant into a different label set and reported accuracies of 0.83–0.85 and macro-F1 values of 0.59–0.76 under all-days and last-day validation; this is included for context only. Study [9] used VOPTICAL_I1 for its main classification analysis and VOPTICAL_Is primarily to examine robustness to illumination and noise, whereas [12] evaluated VOPTICAL_I1 and VOPTICAL_L rather than VOPTICAL_Is. In our implementation, LHS tuned dropout and L2 strength, the learning rates were fixed (1 × 10^−3^ for the head and 1 × 10^−4^ for the expansion channels), and pretrained ImageNet channels remained frozen. The low dispersion across repeated cross-validation folds is encouraging for internal stability, but it does not demonstrate external generalization.

Figure 4 illustrates Grad-CAM and Grad-CAM++ responses for the ShECS binary classifier; Figure 5 presents the same type of qualitative output for the four TFLL categories.

For the TFLL examples in Figure 5, the highlighted areas show where the network accumulated class-specific evidence. We did not compare these maps with expert segmentations, and Tearscope images do not directly visualize meibomian glands. Table 4 summarizes model size and trainability.

### 4.2. Evaluation and Limitations

Several features of the study limit the scope of inference. ShECS contributes a relatively small analytical image set. VOPTICAL-Is contains only healthy, dark-eyed participants aged 19–33 years [19], so its classification task is an image-pattern problem rather than a validation of clinical DED subtypes. Subject identifiers are not documented for VOPTICAL-Is; a numeric code in the file names may correspond to participants, and the diagnostic analyses in Section 2.1 indicate that image-level evaluation is sensitive to this grouping structure. A code-grouped sensitivity analysis was executed on the code-grouped partition: a frozen ImageNet-initialized ResNet-50 feature extractor with a retrained multilayer-perceptron head (seed 42) reached 0.59 held-out accuracy (95% Wilson *CI*: 0.48–0.70), a macro one-versus-rest AUC of 0.85, per-class AUCs of 0.88, 0.85, 0.77, and 0.89, and macro precision, recall, and *F1*-score values of 0.57, 0.58, and 0.57. This performance is substantially below the 0.81–0.90 accuracy range reported across the original VOPTICAL-Is image-level configurations in Table 2. However, the comparison is not a controlled partition-only experiment because the grouped analysis used a frozen ResNet-50 feature extractor whereas Table 2 contains different architectures and trainability policies. The numerical difference therefore should not be interpreted as a direct measure of subject-level leakage or as proof that the file-name codes are participant identifiers. Instead, it demonstrates sensitivity to group-aware partitioning and reinforces the need for identified subject-level cohorts. The eleven Table 2 configurations were not re-executed under code-grouped partitioning; their original image-level ranking is therefore not used to claim code-grouped or subject-level generalization. For the ablation, representative diagnostic figures for seed 42 (training/validation loss and accuracy curves, the 4 × 4 confusion matrix, and one-versus-rest ROC curves) are included as Supplementary Figures S1–S4 in the accompanying figure supplement (each a three-panel diagnostic page showing training/validation loss and accuracy curves, the 4 × 4 confusion matrix, and one-versus-rest ROC curves; one per factorial cell, seed 42), and the complete per-seed metrics for all twelve model runs are provided in Supplementary Table S1 of the Supplementary Material. The four TFLL categories are unevenly represented (159, 117, 90, and 40 images) [19]. In addition, no extensive augmentation was used and neither task was tested in an external multicenter cohort. To address the width-versus-trainability confound directly, a controlled 2 × 2 factorial ablation was executed on the code-grouped partition in TensorFlow/Keras with a reconstructed ResNet-50 backbone: channel width (standard vs. widened ×3) was crossed with trainability policy (frozen backbone vs. trainable conv5 stage) (the four cells are defined explicitly in Section 2.1: the frozen cells keep the pretrained backbone frozen and train the classification head, plus the expansion branch in the widened variant; the conv5-trainable cells additionally train the backbone’s final conv5 stage) while the partition (293 training, 32 validation, and 81 held-out test images), preprocessing, optimization budget, and held-out images were held fixed, with three seeds per cell and the same 81-image held-out set reused in every seed. Table 5 reports the descriptive mean (SD across seeds) results; in a SHA-256 duplicate audit no exact duplicate was found between the development and held-out test sets, and the only exact duplicates were two pairs within the held-out test set; after retaining one deterministic representative per pair, the resulting 79-image sensitivity set gave closely matching values (duplicate-excluded accuracy 0.468 vs. 0.469 for the frozen standard-width cell). Descriptively, unfreezing the conv5 stage was associated with higher accuracy and macro-F1 in both width variants (standard width: accuracy 0.556 vs. 0.469; macro-F1 0.490 vs. 0.410). However, the marginal effect of widening was small and direction-inconsistent across metrics and policies, and the width-by-trainability interaction contrasts were negative on average with seed-to-seed SDs larger than the means. These are descriptive summaries across three seeds; no significance testing was performed, and no generalized ranking of the eleven Table 2 configurations is implied. Because the original custom training source is unavailable, the ablation used a TensorFlow/Keras ResNet-50 reconstruction rather than an exact re-execution of the original custom pipeline. Its inference is therefore limited to this reconstruction: it does not test whether the observed effects transfer to the original ResNet-101×3 pipeline, its absolute values are not directly comparable with the single-seed grouped run above or with Table 2, and it does not constitute evidence about subject-level leakage. The <2% repeated-CV variation supports internal consistency only. Future studies should use identified subject-level partitions, independent cohorts, calibration analyses, clinically plausible augmentation, and prospective evaluation. The held-out sets (87 ShECS and 81 VOPTICAL-Is images) support a proof-of-concept comparison, and the reported confidence intervals quantify the resulting uncertainty; differences between architectures in Table 1 and Table 2 are therefore interpreted as descriptive rather than conclusive.

### 4.3. Scalability and Interoperability

Table 4 highlights the computational cost of widening: total parameter count rises substantially, while only the added filters and MLP head are updated in the widened models. Because the baseline and widened networks in Table 4 follow different trainability policies, that comparison is not a controlled width-only ablation; the independent descriptive effects of width and trainability are instead addressed by the controlled 2 × 2 factorial ablation reported in Section 4.2 (Table 5). For reference, the analytically computed cost of the unmodified backbones at the 384 × 384 input size is 12.0 GMACs for ResNet-50, 22.9 GMACs for ResNet-101 and 33.8 GMACs for ResNet-152 (convolutional layers only). Under the same analytic accounting, the widened variants cost approximately 36 GMACs (ResNet-50×3), 69 GMACs (ResNet-101×3) and 135 GMACs (ResNet-152×4), because the added expansion filters increase the output channels of each convolution by the width factor while their input channel depth is unchanged. These are analytical approximations; measured FLOPs/MACs, latency and memory were not profiled.

Deployment questions were outside the present experiments. We did not measure production inference latency, EHR or JSON interoperability, federated-learning behavior, or operational teleophthalmology performance. Before deployment, future work should report model size, memory demand, FLOPs/MACs, hardware-specific latency, calibration, robustness of preprocessing across imaging devices, and performance in multicenter clinical workflows.

## 5. Conclusions

This study evaluated a deep learning workflow that combined channel expansion with differential freezing/training and qualitative post hoc explanation in two separate ophthalmic imaging problems. Under the original evaluations, the ResNet-101×3 configuration achieved 74% accuracy for ShECS DED classification and the highest numerical accuracy in the original VOPTICAL-Is image-level comparison (90%, macro-F1 = 0.89). Grad-CAM and Grad-CAM++ supplied qualitative visualizations of model attention only; they were not clinically validated. Because VOPTICAL-Is comprises healthy subjects, and subject identifiers are not documented in the accessed release, subject overlap cannot be excluded. A separate code-grouped sensitivity analysis using a frozen ResNet-50 feature extractor with a retrained head yielded 0.59 held-out accuracy. Because this grouped run did not re-run an identical original configuration, the numerical difference from the original image-level results cannot be attributed solely to partitioning and does not directly quantify subject-level leakage. Rather, it demonstrates sensitivity to group-aware partitioning. The original VOPTICAL-Is image-level results should therefore be treated as exploratory, and not as evidence of subject-level generalization. In addition, the relative contribution of channel widening versus freezing/fine-tuning was examined in a controlled 2 × 2 factorial ablation under the code-grouped partition, in which unfreezing the final stage was associated descriptively with higher accuracy and macro-F1 while the effect of widening was small and inconsistent across policies; this ablation used a TensorFlow/Keras ResNet-50 reconstruction rather than an exact re-execution of the original pipeline, and its across-seed summaries are descriptive (no significance testing). These preliminary proof-of-concept findings require validation in larger, identified, subject-level, multicenter, and prospective cohorts before any clinical or teleophthalmology use.

## Figures and Tables

**Table 1:** Binary DED classification (ShECS, held-out test 87 images). Accuracy is reported with 95% Wilson score intervals; precision, recall, *F1*-score, and AUC are point estimates. DED is the positive class.

Architecture	*Accuracy* (95% *CI*)	*Precision*	*Recall*	*F1*-Score	AUC
Rs50 1k-1	0.70 (0.60–0.79)	0.66	0.66	0.66	0.66
Rs50 21k-1	0.71 (0.61–0.80)	0.67	0.66	0.66	0.66
Rs101 1k-1	0.66 (0.55–0.75)	0.33	0.50	0.40	0.33
Rs101 21k-1	0.74 (0.63–0.82)	0.78	0.64	0.70	0.66
Ef B0 21k-1	0.66 (0.55–0.75)	0.33	0.48	0.39	0.48
Rs50×3 1k-1	0.72 (0.62–0.81)	0.71	0.62	0.66	0.61
Rs50×3 21k-1	0.72 (0.62–0.81)	0.79	0.59	0.68	0.59
**Rs101×3 1k-1**	**0.74 (0.63–0.82)**	**0.80**	**0.90**	**0.85**	**0.80**
**Rs101×3 21k-1**	**0.74 (0.63–0.82)**	**0.80**	**0.90**	**0.85**	**0.80**
Rs152×4 1k-1	0.71 (0.61–0.80)	0.67	0.67	0.67	0.67
Rs152×4 21k-1	0.66 (0.55–0.75)	0.33	0.50	0.40	0.33

Note: Bold indicates the best-performing configuration(s) based on the overall reported metric profile.

**Table 2:** Original image-level four-class TFLL interference pattern classification (VOPTICAL-Is, held-out test: 81 images). Accuracy is reported with 95% Wilson score intervals; precision, recall, and *F1*-score are macro-averages; AUC is computed from held-out predicted probabilities. These values describe the original image-level comparison and should not be interpreted as code-grouped or subject-level generalization.

Architecture	*Accuracy* (95% *CI*)	*Precision*	*Recall*	*F1*-Score	AUC
Rs50 1K-2	0.86 (0.77–0.92)	0.89	0.86	0.87	0.97
Rs50 21K-2	0.86 (0.77–0.92)	0.88	0.87	0.87	0.96
Rs101 1K-2	0.85 (0.76–0.91)	0.85	0.85	0.85	0.97
Rs101 21K-2	0.85 (0.76–0.91)	0.85	0.82	0.83	0.94
Rs50×3 1K-2	0.86 (0.77–0.92)	0.89	0.88	0.88	0.97
Rs50×3 21K-2	0.86 (0.77–0.92)	0.89	0.86	0.87	0.97
**Rs101×3 1K-2**	**0.90 (0.82–0.95)**	**0.89**	**0.90**	**0.89**	**0.98**
Rs101×3 21K-2	0.86 (0.77–0.92)	0.89	0.87	0.88	0.97
Rs152×4 1K-2	0.81 (0.72–0.88)	0.86	0.78	0.82	0.96
Rs152×4 21K-2	0.86 (0.77–0.92)	0.88	0.86	0.87	0.97
Ef B0 21K-2	0.81 (0.72–0.88)	0.84	0.76	0.80	0.94

Note: Bold indicates the best-performing configuration(s) based on the overall reported metric profile.

**Table 3:** Contextual comparison with earlier TFLL classification studies.

Ref	Dataset	Method	Best Reported Result	Important Comparison Note/Post Hoc Explanation
[9]	VOPTICAL_I1: 105 images (optimum illumination); VOPTICAL_Is: 406 images (varying illumination)	Color/texture descriptors + feature selection + SVM	97.14% on VOPTICAL_I1; 93.84% robustness result on VOPTICAL_Is	VOPTICAL_Is was used to assess robustness to illumination/noise; the dataset comprised healthy dark-eye subjects. No post hoc explanation.
[10]	105 images from healthy subjects; four Guillon classes	Texture analysis in three color spaces + multiple classifiers	Maximum accuracy > 95%	10-fold CV; four-class TFLL pattern classification. No post hoc explanation.
[11]	105 images from healthy subjects; four Guillon classes	Color/texture features + PCA	Classification rate > 95% in some configurations	Feature-engineered approach on a small image set. No post hoc explanation.
[12]	VOPTICAL_I1: 105 dark-eye images; VOPTICAL_L: 108 light-eye images; combined set	Feature selection + multiple classifiers including libSVM	94.29% dark; 91.67% light; 90.14% combined	Different variants from VOPTICAL_Is; evaluates eye-color/generalization effects. No post hoc explanation.
[29]	VOPTICAL_I1: 105 images; VOPTICAL_L: 108 images	EfficientNet-B0 + MobileNetV2 deep features + Marine Predator feature selection + KNN	98.08% (VOPTICAL_I1); 98.15% (VOPTICAL_L)	Different variants from VOPTICAL_Is; random 50:50 image-level partition; also reports precision, recall, *F1*-score and kappa. No post hoc explanation.
[30]	89,033 image patches from one healthy participant (smartphone-based interferometry, four days)	Lab color + GLCM texture features with a machine-learning classifier	Accuracy 0.853, macro-F1 0.755 (all-days fold); accuracy 0.829, macro-F1 0.587 (last-day fold)	Different label set (colorful, grayish, transparent, non-ROI), device and unit of analysis (patches); not directly comparable. No post hoc explanation.
Ours	ShECS: 434 images; VOPTICAL_Is: 406 images	Widened ResNet/EfficientNet + differential training + LHS	74% DED detection; 90% TFLL pattern classification	VOPTICAL_Is used an image-level split because subject IDs were unavailable; Grad-CAM/Grad-CAM++.

**Table 4:** Model size and trainability of the evaluated backbones.

Model	Total Parameters	Trainable Parameters	Width Multiplier	Frozen Layers	Final Block Channels
ResNet-50 (Baseline)	23,587,714	23,587,714	×1	None	2048
ResNet-101×3	132,541,188	10,488,324	×3	Base blocks frozen; added channels and MLP-trained	6144
ResNet-152×4	260,119,556	16,517,124	×4	Base blocks frozen; added channels and MLP-trained	8192
EfficientNet-B0 (Baseline)	~5.3 M	~5.3 M	Compound scaling	None	1280

**Table 5:** Controlled 2 × 2 factorial ablation on the code-grouped partition (TensorFlow/Keras ResNet-50 reconstruction; 293 training, 32 validation, and 81 held-out test images; three seeds per cell with the same held-out set). Accuracy, macro-averaged precision/recall/F1, and macro one-versus-rest AUC are means with SD across seeds (SD in parentheses); the last two columns give the 79-image exact-duplicate-excluded sensitivity values.

Width	Trainability	Accuracy	Prec.	Rec.	Macro-F1	OVR-AUC	Dup-79 acc.	Dup-79 F1
Standard	Frozen	0.469 (0.074)	0.440 (0.106)	0.445 (0.065)	0.410 (0.113)	0.813 (0.009)	0.468 (0.076)	0.411 (0.115)
Standard	Conv5 trainable	0.556 (0.086)	0.511 (0.089)	0.512 (0.076)	0.490 (0.106)	0.845 (0.019)	0.553 (0.082)	0.491 (0.102)
Widened ×3	Frozen	0.481 (0.057)	0.485 (0.090)	0.488 (0.070)	0.431 (0.098)	0.826 (0.005)	0.481 (0.058)	0.432 (0.099)
Widened ×3	Conv5 trainable	0.514 (0.019)	0.503 (0.025)	0.486 (0.020)	0.457 (0.028)	0.837 (0.015)	0.515 (0.019)	0.461 (0.028)

Values are descriptive means with SDs across the three seeds; the same 81-image held-out set is reused across all seeds, and no significance testing was performed. Dup-79 denotes the 79-image exact-duplicate-excluded sensitivity set.

## Data Availability

VOPTICAL-Is (VOPTICAL_Is) can be obtained through the VARPA research-database access process [19]. The ShECS slit lamp subset used in this analysis is not openly released. Requests may be directed to the corresponding author and require data transfer authorization from the Ophthalmic Epidemiology Research Center, Shahroud University of Medical Sciences.

## Supplementary Materials

The following supporting information can be downloaded at: https://www.mdpi.com/article/10.3390/diagnostics16213569/s1, Table S1: Per-seed performance of the four 2 × 2 factorial-ablation configurations on the VOPTICAL-Is code-grouped held-out test set (12 model runs). Figure S1: Seed 42, standard width, fully frozen backbone (factorial cell: standard_frozen). Diagnostic panels show training and validation loss and accuracy versus epoch, the 4 × 4 held-out confusion matrix, and one-versus-rest ROC curves. Figure S2: Seed 42, standard width, conv5-stage trainable (factorial cell: standard_conv5_trainable). Diagnostic panels show training and validation loss and accuracy versus epoch, the 4 × 4 held-out confusion matrix, and one-versus-rest ROC curves. Figure S3: Seed 42, widened ×3, fully frozen backbone (factorial cell: wide3_frozen). Diagnostic panels show training and validation loss and accuracy versus epoch, the 4 × 4 held-out confusion matrix, and one-versus-rest ROC curves. Figure S4: Seed 42, widened ×3, conv5-stage trainable (factorial cell: wide3_conv5_trainable). Diagnostic panels show training and validation loss and accuracy versus epoch, the 4 × 4 held-out confusion matrix, and one-versus-rest ROC curves. A separate supplementary CSV file contains the file-level code-grouped split assignments for all 406 VOPTICAL-Is images.
