# Supplementary material for: A Deep Learning Framework with Qualitative Post Hoc Explanation for Dry Eye Detection and Tear Film Lipid Layer Pattern Classification Using Anterior-Segment and Tearscope Imaging
Source: Diagnostics (Basel). 2026 Oct 29;16(21):3569. doi: 10.3390/diagnostics16213569 (PMC13762207; doi:10.3390/diagnostics16213569)
Supplement: Supplementary file 1 [file diagnostics-16-03569-s001.zip › diagnostics_manuscript_supplementary.pdf]

Supplementary Materials

Manuscript: A Deep Learning Framework with Qualitative Post-Hoc Explanation for Dry Eye Detection and Tear-Film Lipid-Layer Pattern Classification Using Anterior-Segment and Tearscope Imaging

Table S1. Per-seed performance of the four 2 × 2 factorial-ablation configurations on the VOPTICAL-Is code-grouped held-out test set (12 model runs). Each factorial cell (channel width: standard vs. widened ×3; trainability policy: frozen backbone vs. trainable conv5 stage) was trained with three random seeds (42, 123, 2026) on the code-grouped partition (293 training, 32 validation, and 81 held-out test images). Accuracy is reported with the 95% Wilson score confidence interval; macro-precision, macro-recall, macro-F1, and macro one-versus-rest AUC are computed over the four tear-film lipid-layer classes. Dup-79 columns refer to the 79-image exact-duplicate-excluded sensitivity set.

Factorial cell	Seed	Accuracy (95% CI)	Macro-precision	Macro-recall	Macro-F1	Macro OVR-AUC	Dup-79 acc.	Dup-79 F1
standard_frozen	42	0.469 (0.364–0.577)	0.440	0.445	0.410	0.813	0.468	0.411
standard_frozen	123	0.543 (0.435–0.647)	0.546	0.510	0.523	0.822	0.544	0.526
standard_frozen	2026	0.395 (0.296–0.504)	0.334	0.380	0.297	0.804	0.392	0.296
standard_conv5_trainable	42	0.556 (0.448–0.659)	0.511	0.512	0.490	0.845	0.553	0.491
standard_conv5_trainable	123	0.642 (0.533–0.738)	0.600	0.588	0.596	0.864	0.635	0.593
standard_conv5_trainable	2026	0.470 (0.365–0.578)	0.422	0.436	0.384	0.826	0.471	0.389
wide3_frozen	42	0.481 (0.376–0.588)	0.485	0.488	0.431	0.826	0.481	0.432
wide3_frozen	123	0.538 (0.430–0.642)	0.575	0.558	0.529	0.831	0.539	0.531
wide3_frozen	2026	0.424 (0.322–0.533)	0.395	0.418	0.333	0.821	0.423	0.333
wide3_conv5_trainable	42	0.514 (0.407–0.620)	0.503	0.486	0.457	0.837	0.515	0.461
wide3_conv5_trainable	123	0.533 (0.425–0.638)	0.528	0.506	0.485	0.852	0.534	0.489
wide3_conv5_trainable	2026	0.495 (0.389–0.602)	0.478	0.466	0.429	0.822	0.496	0.433

Values are descriptive per-seed results; the same 81-image held-out set is reused across all seeds and no significance testing was performed. The mean (SD) across the three seeds of each cell corresponds to the values reported in Table 5 of the main text.

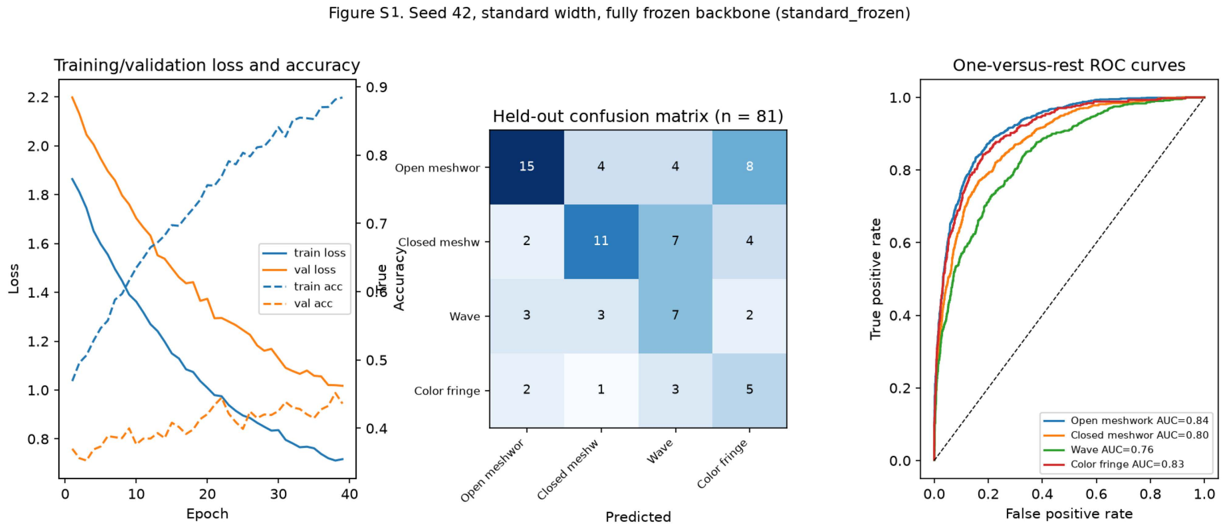

Figure S1. Seed 42, standard width, fully frozen backbone (factorial cell: standard_frozen). Diagnostic panels show training and validation loss and accuracy versus epoch, the 4 × 4 held-out confusion matrix, and one-versus-rest ROC curves.

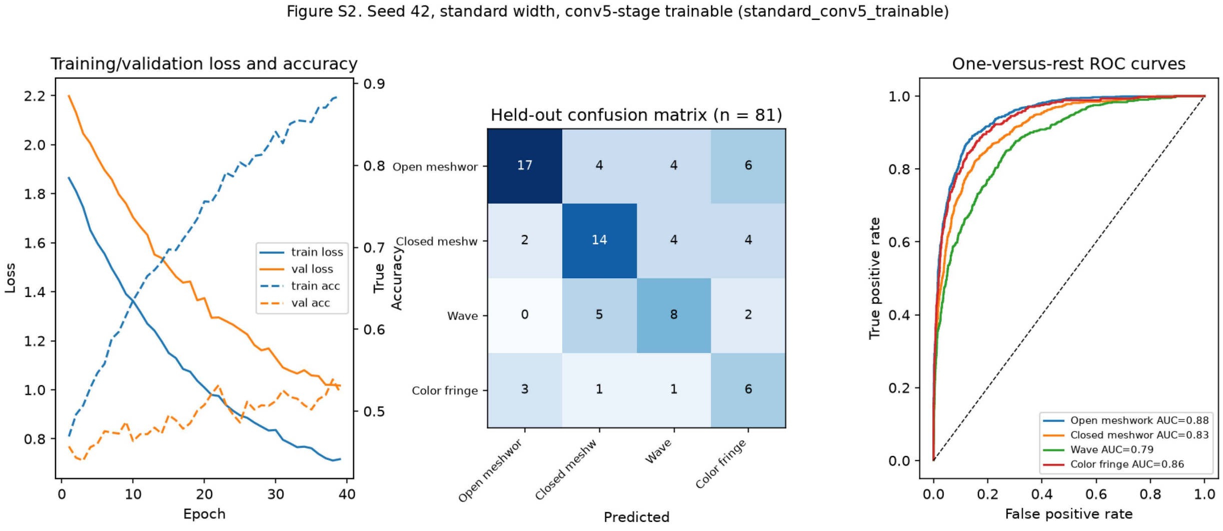

Figure S2. Seed 42, standard width, conv5-stage trainable (factorial cell: standard_conv5_trainable). Diagnostic panels show training and validation loss and accuracy versus epoch, the 4 × 4 held-out confusion matrix, and one-versus-rest ROC curves.

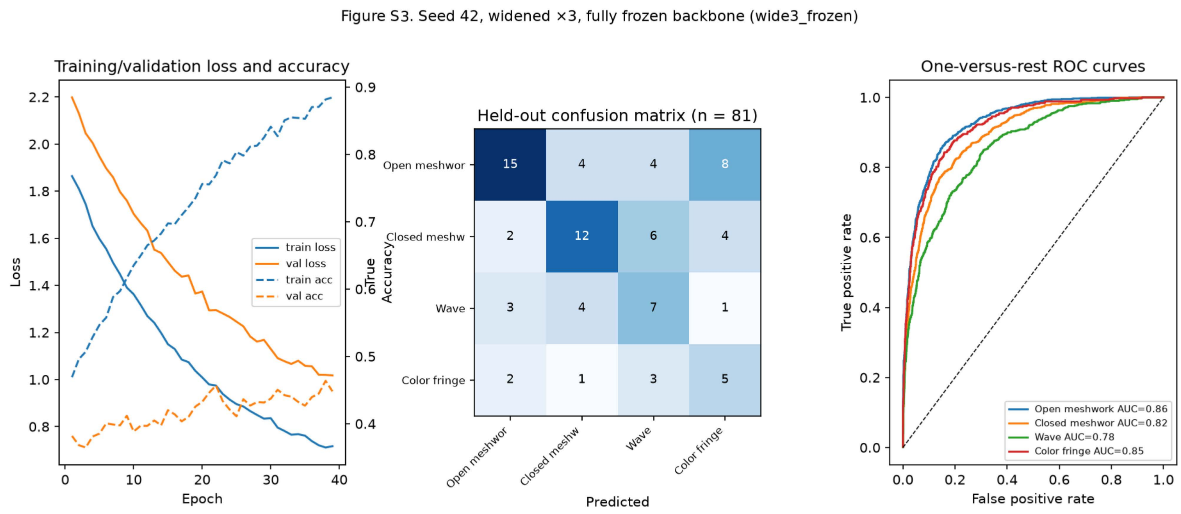

Figure S3. Seed 42, widened ×3, fully frozen backbone (factorial cell: wide3_frozen). Diagnostic panels show training and validation loss and accuracy versus epoch, the 4 × 4 held-out confusion matrix, and one-versus-rest ROC curves.

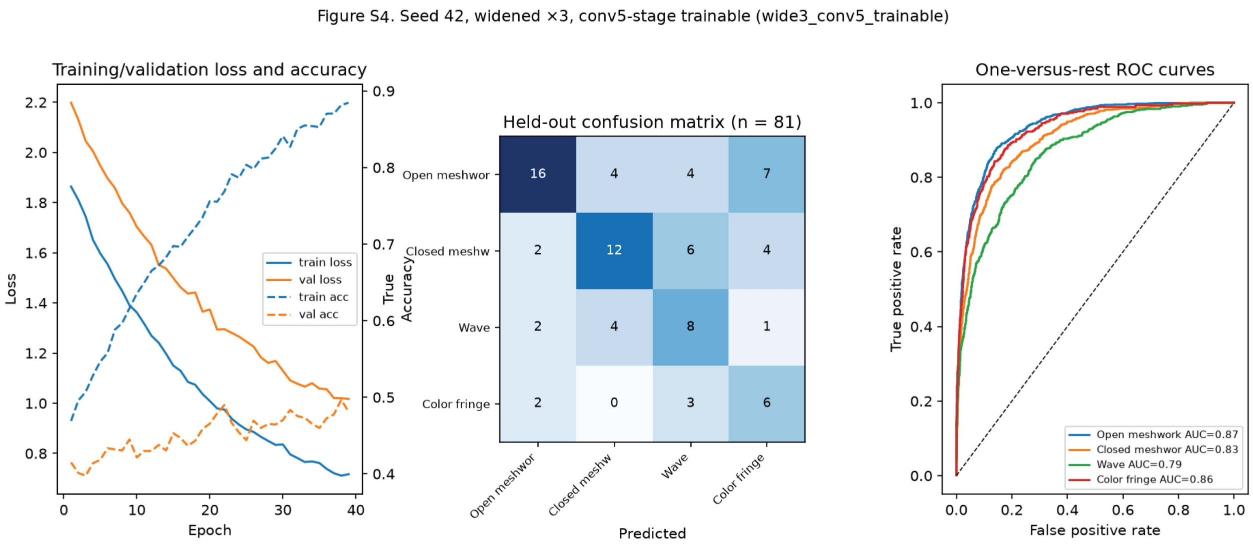

Figure S4. Seed 42, widened ×3, conv5-stage trainable (factorial cell: wide3_conv5_trainable). Diagnostic panels show training and validation loss and accuracy versus epoch, the 4 × 4 held-out confusion matrix, and one-versus-rest ROC curves.

Supplementary CSV file

The supplementary file **VOPTICAL_Is_code_grouped_split.csv** (provided separately) contains the file-level code-grouped split assignment for all 406 VOPTICAL-Is images: file name, participant code, split label (train / validation / test), and the annotated Guillon tear-film lipid-layer class (open meshwork, closed meshwork, wave, color fringe). The four held-out codes are 002, 005, 011, and 016 (81 images); the validation codes are 003, 007, and 012 (32 images); the remaining 293 images belong to 10 training codes.
